# Psychophysical Evaluation of Sensory Reweighting in Bilateral Vestibulopathy

**DOI:** 10.3389/fneur.2018.00377

**Published:** 2018-05-25

**Authors:** W. Pieter Medendorp, Bart B. G. T. Alberts, Wim I. M. Verhagen, Mathieu Koppen, Luc P. J. Selen

**Affiliations:** ^1^Radboud University Nijmegen, Donders Institute for Brain, Cognition and Behaviour, Nijmegen, Netherlands; ^2^Department of Neurology, Canisius Wilhelmina Hospital, Nijmegen, Netherlands

**Keywords:** spatial orientation, vertical perception, multisensory integration, sensory reweighting, rod-and-frame, bilateral vestibular areflexia, psychophysics, Bayesian integration

## Abstract

Perception of spatial orientation is thought to rely on the brain’s integration of visual, vestibular, proprioceptive, and somatosensory signals, as well as internal beliefs. When one of these signals breaks down, such as the vestibular signal in bilateral vestibulopathy, patients start compensating by relying more on the remaining cues. How these signals are reweighted in this integration process is difficult to establish, since they cannot be measured in isolation during natural tasks, are inherently noisy, and can be ambiguous or in conflict. Here, we review our recent work, combining experimental psychophysics with a reverse engineering approach, based on Bayesian inference principles, to quantify sensory noise levels and optimal (re)weighting at the individual subject level, in both patients with bilateral vestibular deficits and healthy controls. We show that these patients reweight the remaining sensory information, relying more on visual and other nonvestibular information than healthy controls in the perception of spatial orientation. This quantification approach could improve diagnostics and prognostics of multisensory integration deficits in vestibular patients, and contribute to an evaluation of rehabilitation therapies directed toward specific training programs.

## Introduction

Accurate perception of gravity is important for spatial orientation, the maintenance of balance, and the regulation of gait. While the vestibular sense is crucial, it is known that visual, proprioceptive, and somatosensory signals are also used and integrated to estimate the gravitational direction (1–3). In addition, cognitive processes and inflows have been suggested to contribute to deriving this estimate (4, 5). When one of these signals breaks down because of injury, disease, or aging, perception of gravity is disturbed, which can result in inability to orient correctly, reduced ability to stand or walk, and even falling (6–8).

Such sensory impairments not only have a huge impact on quality of life and productivity but also impose high costs to public health service (9, 10). In Europe, for example, more than 20% of the population will be over 65 in 2025, with a particularly rapid increase in the number of persons over 80, and many of them showing age-related functional sensory loss (11). Sensory dysfunction also affects members of the younger population, e.g., through genetic disposition [such as Usher syndrome, see Ref. (12)], as a result of accidents, or through work-related exposure to harmful sensory stimuli, and this represents a significant economic burden to society. Minimizing the impact of sensory impairments is, therefore, important from various perspectives.

Sensory impairments, while debilitating, may be difficult to diagnose for a number of reasons. First, it is not straightforward to measure the various contributing sensory systems in isolation during natural tasks. For example, a tilt of the head is not only sensed by the vestibular organs, located in the inner ear, but also by the proprioceptors in the neck. But then, one cannot switch off the proprioceptive sense and measure just the vestibular sense in natural conditions. Second, different sensory systems have different dynamics and sometimes provide conflicting information, e.g., visual cues can conflict with vestibular cues. Third, sensory signals can be ambiguous; e.g., the otoliths cannot discriminate between gravity and other linear accelerations (13). Fourth, sensory signals are inherently noisy, which makes them unreliable to some extent by definition; in fact, their noise level is not even fixed, but may, for example, depend on the signal’s strength (14, 15). Finally, if one signal deteriorates, or breaks, remaining senses can compensate for this loss; this process, called *sensory reweighting*, is useful, but masks a direct view on the origin of the sensory deficit. Sensory integration reflects the interplay of all these factors, which in turn, makes it difficult to decompose this process into its constituent elements.

Most standard vestibular tests address reflexive behaviors rather than the natural behaviors that depend on the integration of multiple sensory signals (8). For example, tests such as the head impulse test, the caloric test, or VEMP testing merely probe the vestibular system in isolation, in an open-loop manner. While these tests make important contributions to vestibular diagnosis, they lack the sensitivity and selectivity to reveal the weighting of the vestibular component in multisensory integration. Also, the Romberg test and other dynamic posturography tests are difficult to interpret when it comes to a precise quantification of how vestibular signals contribute to the sensory integration process (8).

There is a considerable potential for new diagnostics and prognostics approaches on deficits in multisensory integration (16). Such approaches should aid in tracking the quality of sensory systems across the life span or disease, addressing the risk factors, and signaling when (older) people and patients may be in need of additional care or training programs to keep living an active life. Prognostic and diagnostic markers of the underlying sensory deficits could help in developing programs that mitigate risks for these and other people.

In the present paper, we describe a novel psychophysical approach to assessing sensory reweighting in bilateral vestibular patients. This approach culminated from a series of modeling and psychophysics studies that we performed over recent years to understand the integration of the multiple sensory cues for spatial orientation (5, 17–22). Recently, all this work has been extensively reviewed by Kheradmand and Winnick (23) and we refer the reader there for an overview.

In the present paper, we focus on the use of a reverse engineering approach for assessing multisensory integration and reweighting in bilateral vestibular patients. We first provide a short summary of our approach and what it has revealed about sensory integration in healthy participants. Next, we will demonstrate the utility of this approach for clinical testing, showing that it explains major task-dependent features as well as idiosyncratic differences of bilateral vestibular patients in spatial orientation tasks.

## Statistical Framework

Sense organs, for instance, those informing the brain about the position or orientation of body or body parts, have only limited precision. The same physical situation will, across different instances, lead to similar, but not identical neural firing patterns. Conversely, one particular neural firing pattern of a sense organ may, in different instances, result from resembling, but not identical physical situations. Due to the omnipresence of such sensory noise, the input–output relationship is not deterministic, but rather probabilistic in character, even in the absence of sensory ambiguities or conflicts (24).

This means that for modeling the information transfer from sensory inputs to the state estimate inferred a probabilistic approach is called for. That is, the output of an individual sensory source is not taken to be one specific state estimate, but rather a probability distribution of state estimates (often a Gaussian distribution is assumed) centered at some state, but with a certain amount of spread. This spread, the variance of the distribution, represents the sensory noise level. The statistically optimal strategy for achieving a state estimate from multiple probabilistic sensory signals is known as Bayesian integration. In this framework, uncertainty about the state is reduced by fusing overlapping sensory information, weighting each sensory signal in proportion to its reliability, i.e., inversely proportional to its noise level (25–27).

Various perceptual studies have provided evidence that the brain might perform such Bayesian multisensory integration. The approach of these studies was to first estimate the noise levels of the individual sensory sources and then use these isolated measures to predict performance in the combined condition (28). Unfortunately, such a forward approach cannot be applied when the contributing signals cannot be assessed in isolation, as in spatial orientation, which is based on visual, somatosensory, and vestibular cues, as well as cognitive processes.

In Clemens et al. (5), we, therefore, approached this problem from the opposite perspective. We assumed that the behavioral outcomes result from an optimal integration process of multiple sensory modalities and implemented an inverse probabilistic approach to infer, given this assumption, how the individual sensory modalities are weighted in. More specifically, we deduced the individual sensory noise levels by behaviorally probing two state estimates—the orientation of the body-in-space and the orientation of the head-in-space—which, under the assumption of optimal integration, weigh all available sensory signals based on their noise levels, after converting them into the task-specific reference frame.

**Figure 1:**
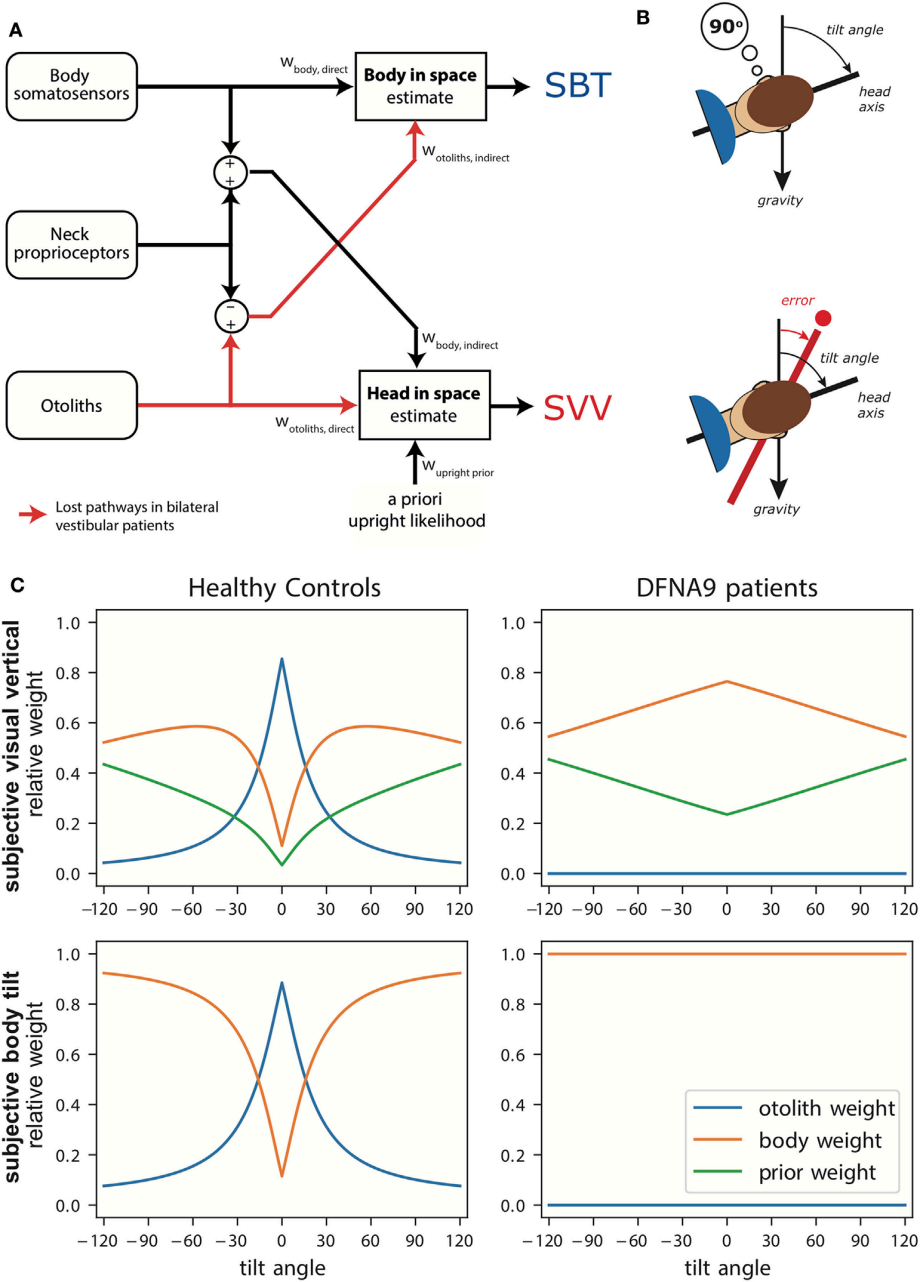
**(A)** Schematic of the Bayesian optimal integration model by Clemens et al. (5). In the subjective body tilt (SBT) task, body somatosensors provide direct information about body orientation in space, whereas the otolith signals undergo a coordinate transformation based on neck proprioception and provide an indirect measurement of body orientation in space. Both signals are weighted (w_body, direct_, w_otoliths, indirect_) based on their reliability to provide an estimate of body orientation in space. Weights are always relative, summing to unity. In the subjective visual vertical (SVV) task, the otoliths provide direct information about the head-in-space and the body somatosensors provide indirect information. In addition, the brain assumes that upright head orientations are more likely based on prior experience. All three signals are weighted (w_otoliths, direct_, w_body, indirect_, w_upright, prior_) in proportion to their reliability to provide a head-in-space estimate. **(B)** Schematic of the SBT and SVV task. In the SBT task, the subject is given a reference orientation (here 90°) and subsequently rotated in roll, in complete darkness, to an orientation around this reference. Subsequently, the subject is asked to indicate whether his/her current orientation is clockwise or counterclockwise relative to the given reference. In the SVV task, the subject is rotated to a roll tilt angle in complete darkness. Subsequently, a line if briefly flashed and the subject must indicate whether this line is oriented clockwise or counterclockwise relative to gravity. **(C)** Weights attributed to the signals in the Bayesian optimal integration model for healthy controls and bilateral vestibular patients for the SVV and SBT task. Note the clear difference in weighting between patients and control. Weights are derived based on sensory noise levels determined in Alberts et al. (20, 22).

Figure 1A illustrates the transformation and integration steps involved in computing the body-in-space and head-in-space estimates. The scheme is based on the processing of signals from three sensory systems: (1) the otoliths, detecting the orientation of the head with respect to gravity; (2) body somatosensory signals, which are sensitive to the orientation of the body-in-space; and (3) neck sensors, which signal the angle between head and body based on proprioception. All sensory signals are taken to be unbiased, but corrupted with independent Gaussian noise with a given variance.

According to the scheme, an estimate of body orientation in space can be obtained directly from body somatosensory signals, but also indirectly from the head-centered otolith signal, by subtracting the head-on-body signal derived from neck proprioception (5). Likewise, the estimate of head-in-space orientation can be obtained directly from the otoliths, but also through an indirect pathway, by combining the body somatosensory signals with neck proprioceptive information (5). Because the body-in-space and the head-in-space estimates require different coordinate system transformations, the noise levels of the direct and indirect pathways, and thus their weighting, differ. Furthermore, a loss or severe disruption of the otolith or somatosensory inputs will deteriorate both state estimates, but should not completely break down performance due to their multimodal dependence on the direct and indirect pathways (5).

In addition to the two sensory pathways, the scheme allows for the possibility that the two orientation estimates are influenced by prior beliefs about certain orientations. In particular, it has been suggested that the brain takes into account in the integration process that the head is oriented in an upright position most of our daily life (4, 17).

Thus, based on optimality principles, the model estimates the noise levels of the involved sensory systems from behavioral responses in tasks that psychometrically probe body-in-space and head-in-space orientation. These noise levels, in turn, determine the relative weight to be attributed to each sensory signal in the integration process. In the same manner, it can be computed how signals are re-evaluated, i.e., reweighted, in the integration process when one of these signals loses fidelity (i.e., becomes noisier), such as in vestibulopathy. For clarity, we note that sensory substitution is also a form of sensory reweighting in that the weight is zero for the lost sense (because it is completely unreliable). Sensory substitution is used in case of complete loss, and may even suggest that this modality was not used before the sensory loss. In this review we use sensory reweighting as the more general term, embracing sensory substitution.

## Measuring Spatial Orientation

To test this model experimentally, it is important to use tasks with outcome measures that allow back-inferring the weights. Two important tasks that are typically used to study spatial orientations are the subjective visual vertical (SVV) and the subjective body tilt (SBT) task [Figure 1B, (5)]. In the SVV task, subjects have to report their perception of the orientation of a visual line relative to the gravitational vertical. Note that, to compute the SVV, the brain not only necessitates an estimate of the orientation of the head-in-space but also must compensate for ocular counterroll (OCR) and its effect on line orientation on the retina. In the SBT task, subjects must report how they perceive the orientation of their body relative to gravity or another given reference angle.

When using these tasks for evaluating sensory reweighting, special attention should be given to how responses are measured. In the literature, various studies tested the SVV and SBT task using adjustment methods [see Ref. (29) for a list of adjustment studies]. For example, (tilted) subjects have to adjust the direction of a visual line in front of them until they perceive it vertical in space. While such adjustment methods are easy and intuitively appealing, doubts about the observer’s interpretation of the perceptual criterion, as well as a possible response bias, could confound the interpretation of the results (30).

This has elicited the development of more objective psychophysical approaches, such as the two-alternative forced choice (2AFC) paradigm. Using this paradigm, subjects are to make on every trial a binary decision relative to the perceptual criterion, for example, judging whether the orientation of a briefly flashed line is counterclockwise (CWW) or clockwise (CW) relative to their perceived direction of gravity. If not sure, subjects must guess. So, using 2AFC, one does not directly measure the point of subjective equality (as in adjustment tasks), but collects psychometric data to determine this point as the 50%-point of a binary choice.

Responses in the 2AFC task can then be summarized by fitting a cumulative Gaussian function. In the SBT task, the mean of the Gaussian (the 50%-point) represents the subjective perception of the reference orientation. In the SVV task, it represents the SVV compensation angle (the angle between the apparent visual vertical line and the body axis). The variance of the Gaussian, inversely related to reliability or precision, serves as a measure of the variability of the subject in the tasks. Compared to the abundant literature on SBT and SVV accuracy, data on their perceptual variability are still quite scarce, although this measure is key in assessing sensory (re)weighting.

## Spatial Orientation in Darkness

We have used this psychophysical approach to test healthy human subjects using the SVV and SBT tasks, performed at tilts <120° (5). We found the SBT to be relatively unbiased across the tilt range and the SVV to show substantial biases for tilt angles beyond 60°. The SVV bias is well known in the literature (31–34) and referred to as the A-effect (35). Furthermore, in both tasks, variability became larger with tilt angle, but appeared consistently lower in the SVV.

We used the sensory integration model, described above, to fit both the SBT and SVV data simultaneously (5). To account for the bias in the SVV, the model suggests a contribution of prior knowledge to the integration process, consistent with previous suggestions that the brain has learnt that the head is typically upright in life (4). Given that the SBT is virtually unbiased suggests that this upright prior is not used in its underlying computations. To explain, one could argue that a head prior reduces variability in the SVV, which may be useful for stable visual processing, but at the expense of a bias. Consistent with a mere role of the prior in visual processing, Bortolami et al. (36) reported virtually no bias in the haptically indicated vertical. A bias is also unwarranted for body orientation perception for reasons of balance and postural control, and the brain rather chooses accuracy over precision.

The model fits also confirmed previous suggestions that otolith noise increases with tilt angle. This decreasing reliability with increasing tilt angle (37, 38) may relate to the utricle containing significantly more hair cells than the saccule (39). This arrangement may yield tilt-dependent noise because the utricle senses most effectively head tilts close to upright, whereas the saccule best detects head tilts around 90°.

The sensory noise parameters determine the optimal sensory weights in the integration process. Figure 1C (left panels) shows these weights in healthy subjects as a function of tilt angle. Perhaps surprisingly, the SBT estimate is not dominated by information from the body receptors in the direct pathway, but is actually mainly determined by the indirect pathway, carrying the signals of the otoliths, in the behaviorally important range near upright. Only at larger tilt angles, when the otoliths become less reliable, the body sensors (direct pathway) start to dominate. For the SVV, the pattern of otolith weights is remarkably similar, again reflecting increasing otolith noise. As the otolith contribution becomes smaller, the contributions of the prior and indirect pathway become more apparent.

Can this model, which provides a computational account of sensory weighting in healthy participants, also be applied to infer the ramifications in case of vestibular deficits? To our knowledge, there have been no studies that tested SVV and SBT within the same patients, at multiple tilt angles, and reporting quantitative values of bias and variability. This is not to ignore that already quite some important work has been done studying spatial orientation in vestibular patient groups (40–43).

We recently measured the SVV and SBT in a homogeneous group of bilateral vestibular patients, diagnosed with a DFNA9 mutation (20). DFNA9 is a progressive autosomal dominant vestibulo-cochlear disorder, in which an acidophilic mucopolysaccharide deposit is found in both the cochlea and macula, causing strangulation of the nerve endings (44, 45). Furthermore, these patients show neuroepithelial and neural degeneration in the inner ear (46). The DFNA9 mutation causes hearing impairment and bilateral vestibular function loss, but does not affect the proprioceptive or visual system. We performed several clinical tests to confirm complete loss of vestibular function, including the OCR task, VEMP measurements, caloric tests, and VOR velocity step tests (90 and 250°/s) [see Ref. (20)].

Because these patients have bilaterally lost the otolith pathway, it is conceivable that they have reweighted the contribution of the sensory modalities to the integrated percept of verticality. We, therefore, tested them in darkness to establish how body and neck sensors now contribute to the SVV and SBT computations (20). Patients and a group of age-matched controls were tested in the upright position (0°) and at 90° sideways roll tilt.

The SVV was unbiased when upright, but showed a stronger bias in the patients than controls at 90° tilt. This increased bias can be understood with the model at hand (Figure 1C, right panels): the sensory-derived head tilt estimate is now solely based on the indirect, body somatosensory, pathway because the otolith weight is set to zero, and thus becomes noisier. This increases the relative weight of the prior and its biasing effect becomes more prominent.

The patients’ perception of body tilt (SBT) was unbiased and showed larger variability in both groups at 90°. From the perspective of the model, this increase of perceptual variability with tilt angle in the patients suggests that body somatosensory cues are also contaminated by tilt-dependent uncertainty just like the otoliths [as established in healthy controls (5, 18)]. Recently, we and other research groups found further support for tilt-dependent somatosensory uncertainty using a paradigm that dissociates the orientations of head and body (22, 47). In these experiments, a head-on-body tilt on top of whole body roll tilt was introduced while the percept of vertical was measured. In Alberts et al. (22), we found that the percept of vertical is processed in a head-in-space reference frame, with an increasing bias for larger head-in-space orientations. From the perceptual variability, we inferred that the otoliths contribute more strongly around upright while the body somatosensors make contributions when the body was tilted to larger angles.

The findings in the DFNA9 patients are consistent with previous reports. For instance, Bisdorff et al. (40) showed that bilateral vestibular patients perform quite accurately in the SBT at upright, but are substantially more variable in their responses than normal subjects. Bronstein et al. (41) reported that vestibular patients still compensate for their tilt angle when testing the SVV at 90°, but with a bias about twice as large as in healthy subjects. With our optimal integration model we are now able to explain both observations in terms of sensory reweighting.

## Spatial Orientation in the Light

Hitherto, we have described the integration of vestibular, proprioceptive, and somatosensory information in spatial orientation. To examine this process, participants are typically tested in darkness. But obviously, spatial orientation is a crucial ability that we also need in the light. In the light, visual contextual information from the surrounding environment provides an important cue for spatial orientation, since most common orientations in a naturalistic visual scene are vertical or horizontal (48–50). The brain is known to use this panoramic information as a gravity indicator (51).

**Figure 2:**
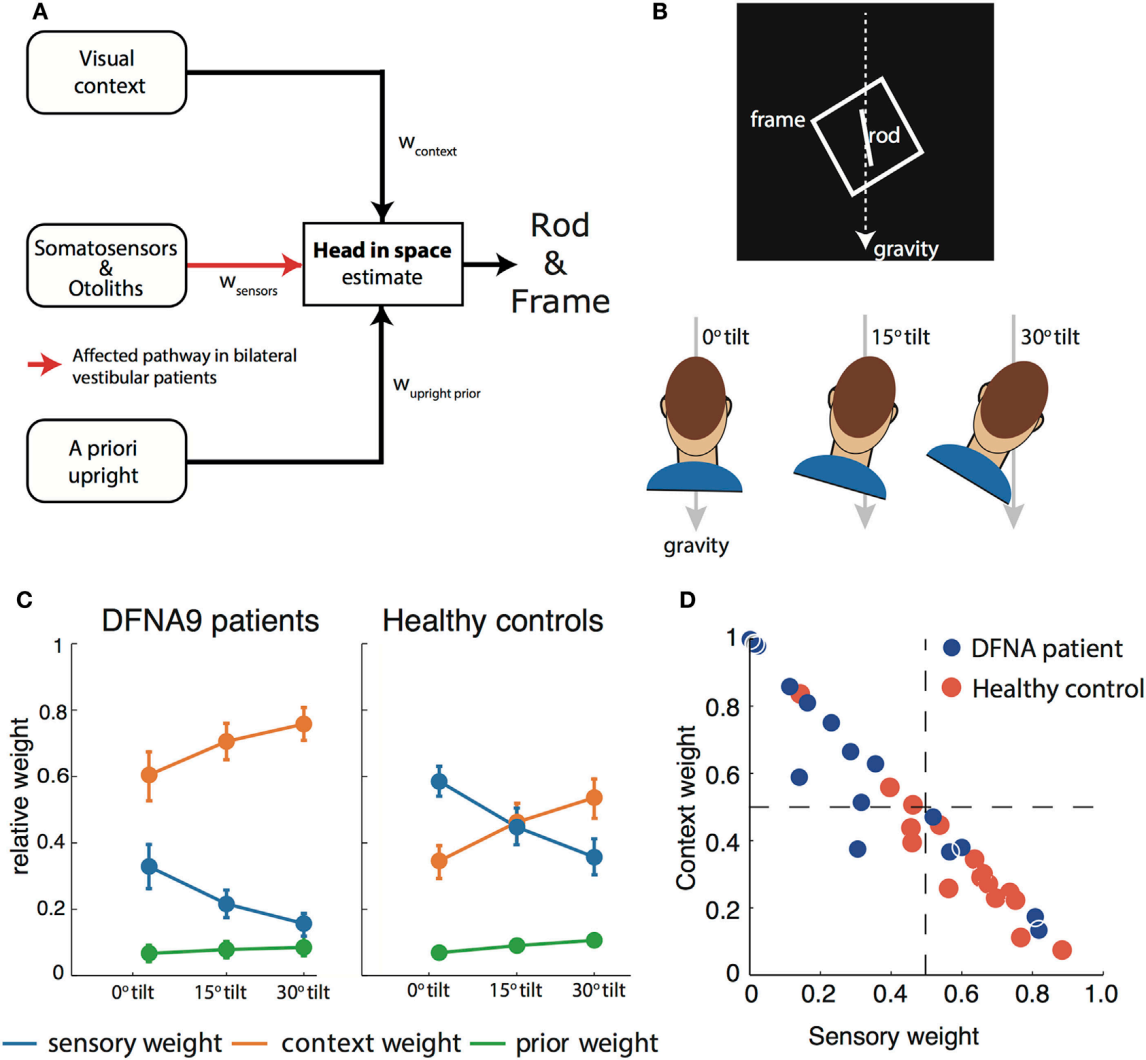
**(A)** Schematic of the Bayesian optimal integration model as proposed in Alberts et al. (21, 55) for the rod-and-frame effect. In the rod-and-frame task, an estimate of head orientation in space is used that is derived from visual context information, sensory information from somatosensors in the body, and otoliths in the head. In addition, the brain assumes that upright positions are more likely based on prior experience. Each of these information streams is weighted into the head-in-space estimate based on its reliability. **(B)** Schematic of the rod-and-frame task. The subject views a rotated frame in which an oriented line is shortly flashed. He/she must indicate whether this line is rotated clockwise or counterclockwise relative to gravity, using a two-alternative forced choice response. Subjects performed this task under three head orientations (0°, 15°, and 30°). **(C)** Weights attributed to the various sources for bilateral vestibular patients and healthy controls. Notice that the patients rely much more (factor 2–3) on the visual contextual information than the controls. **(D)** Weighting of sensory information, lumped from somatosensors and otoliths, and contextual information of the frame to estimate the head orientation in space for the individual patients and controls.

The rod-and-frame task can be used to operationalize the effect of panoramic visual cues on the perception of vertical (52). In the rod-and-frame task (Figure 2B), subjects have to indicate the orientation of a visual line (rod) within a square frame. Previous work has shown that, when seated upright, frames rotated relative to the gravitational vertical cause biases in the rod-and-frame task, showing a periodical modulation. Biases are about absent for upright and ±45° roll-tilted frame orientations, but increase for intermediate frame orientations (52, 53). In Vingerhoets et al. (54), we have shown that these biases increase when the head is tilted, even when the square frame is replaced by a single line in the retinal periphery.

To interpret rod-and-frame effects in terms of optimal sensory integration, a sensory integration model is needed that incorporates visual contextual information. In Vingerhoets et al. (54), we put forward such a model for the first time, structuring how the rod-and-frame effect relates to statistical properties of the various sensory signals that are involved, representing the frame effect as a distribution with four equally high modes corresponding to the orientations of the sides of the square. In Alberts et al. (21), we combined this model with the model of Clemens et al. (5), but lumping the tilt-dependent decrease in precision of the body sensors and otoliths. In addition, we replaced the original four equally high modes relative to the frame with separate modes corresponding to the vertical and horizontal axes of the frame (Figure 2A).

We tested this model by collecting the appropriate psychometric measures—accuracy and variability—in conditions in which we manipulated the frame reliability (by increasing its distance from the observer) and head orientation reliability (by tilting the head). We found that the rod-and-frame effect is reduced when the frame reliability is reduced, meaning that it has a weaker biasing effect, and enhanced when the head is tilted and thus the head orientation signals are reduced in precision. We further found that response variability was lowest when the frame was upright, became greater with larger frame orientations and subsequently leveling off. The sensory integration model, which involves a flexible, precision-dependent weighting of head orientation signals and panoramic visual signals (Figure 2C, right panel), with separate weights for horizontal and vertical panoramic cues, provided a good description of the data. Because the rod-and-frame task, in combination with the integration model, can characterize the weighting of visual and vestibular information in the estimate of verticality, we subsequently also applied it to quantify the visual compensation strategies in bilateral vestibulopathy.

## Sensory Reweighting in Bilateral Vestibulopathy

Recently, we performed a psychophysical evaluation of sensory reweighting in bilateral vestibulopathy using the rod-and-frame task (55). We compared a group of 16 DFNA9 patients to a control group in judging the orientation of a rod (clockwise or counterclockwise relative to gravity), presented within an oriented square frame, while the head was maintained in three different orientations relative to the body. We found larger biases in the patients’ percept of vertical and increased variability compared to the control group.

We then fitted the model to back-infer the noise characteristics of the (remaining) signals and compute the weights from these noise characteristics. This revealed that patients had increased their visual weight by a factor of about 2–3 compared to controls, consistent with the hypothesis that, after vestibular loss, the remaining sensory cues are reweighted (Figure 2C).

A further strength of this psychophysical evaluation is that weights can be determined at the individual level. Most patients manifested a high visual context weight, but in two patients this weight appeared low and rather a high nonvestibular weight bore out (Figure 2D). Such an individualized assessment has good potential for clinical practice, allowing to develop personalized rehabilitation therapies. Healthy controls show a high combined vestibular and nonvestibular weight and are much less influenced by visual contextual cues than the patients.

Based on the findings in our patients, we could interpret the increase of their optokinetic response and their cervico-ocular reflexes (56, 57) as another reflection of the reweighting of visual signals in the remaining integration process. Furthermore, the results of our patients are in harmony with previous studies in bilateral vestibular patients reporting increased reliance on visual cues in spatial orientation tasks (41, 58–63). The added value of our approach, including the computational model, is that all the underlying noise sources could be back-inferred, addressing the increased visual reliance in terms of sensory reweighting and computing the specific sensory weights.

We consider it likely that the reweighting of nonvestibular and visual cues in our patients amounts to sensory substitution in the brain, since our patients showed complete vestibular loss in vestibular diagnostic tests. Of course, increasing the reliance on a visual indicator of what is upright causes a larger bias when the frame axes are not aligned with the gravitational horizontal and vertical. In natural situations, however, this hardly ever happens, which may explain why we found a compensation strategy that enhanced reliance on the visual cues.

The neural correlate of multisensory integration and sensory reweighting remains a matter of speculation. The vestibular nuclei (VN) are the first stage of sensory integration and reweighting for spatial orientation, where neurons are not only tuned to vestibular input, but also to visual, proprioceptive, and motor inputs (3, 64). The VN are structurally and functionally linked to the posterior-temporal junction [TPJ (65)], where the parieto-insular vestibular cortex is situated. Recent brain stimulation studies have implicated the TPJ in estimating the visual vertical (66–68). Other imaging and TMS studies have identified the superior parietal lobule (SPL) for the integration of visual contextual information in the perceived gravity reference frame, mediated by reciprocal inhibitory connections between the early visual areas and the TPJ (67, 69, 70). Thus, if the representation of the gravitational vertical (based on vestibular and nonvestibular signals) is less reliable, there will be more inhibition of the visual contextual representation. This would suggest that visual contextual information drives the SPL more strongly in patients than healthy controls.

## Vestibular Rehabilitation

Using simple tasks, such as the SVV, SBT, and the Rod-and-Frame task, embedded in a psychometric test paradigm, we quantified sensory (re)weighting (or sensory substitution because of complete sensory loss) at the single subject level. Note that we mostly used these tasks in static, sustained conditions [but see Ref. (19)], i.e., when the rotation signals in the canals have died away and the only stimulation to the otoliths is gravity. Assessment of canal contributions in sensory reweighting requires dynamic tasks and measurements, which is outside the scope of this paper.

In particular, the rod-and-frame task appears an effective tool for an individualized assessment of visual–vestibular–somatosensory integration and reweighting. If made clinic-ready, such a task could contribute to prognosticate, diagnose, and evaluate clinical treatment in multisensory integration processes that underlie spatial orientation, postural balance, and regulation of gait. Before reaching this stage, however, various aspects need to be optimized, from stimulus design to data recording, test duration, and data-analysis. One way to proceed is by incorporating modern adaptive psychometric procedures, which could improve efficiency in parameter estimation, both in terms of number of trials needed and the quality of the estimates (71, 72).

To date, most vestibular rehabilitation programs consist of exercises that aim to improve postural stability and visual acuity and decrease complaints of dizziness, visual vertigo, and oscillopsia. For balance training (73), our results may suggest (but this needs to be tested) that patients with a larger visual weight will profit more from using the visual context as a vestibular replacement, whereas patients with low visual weight may gain more from somatosensory training. This weight distribution may change with time, i.e., when the rehabilitation has been effective or when disease progresses.

The presented approach, based on inverse probabilistic modeling, could make vestibular rehabilitation programs more specific and better tailored to the need of the end user or patient, providing information to track the recovery, decline, or disease.

## Author Contributions

WPM wrote the first draft of the manuscript. WPM drafted and LS created the figures. All authors were involved in editing the final version of the manuscript.

## Conflict of Interest Statement

The authors declare that the research was conducted in the absence of any commercial or financial relationships that could be construed as a potential conflict of interest.
